# Feasibility Pilot of the LaceUp Compression–Weight Sleeve for Essential Tremor

**DOI:** 10.3390/bioengineering13070785

**Published:** 2026-07-08

**Authors:** Alessandro Napoli, Hannah Gravante, Tori Hamilton, Nicole Gerhardt, Mijail D. Serruya

**Affiliations:** 1 Raphael Center for Neurorestoration, Vickie & Jack Farber Institute for Neuroscience, Philadelphia, PA 19107, USA; mijail.serruya@jefferson.edu; 2 Center for Outcomes & Measurement, Jefferson College of Rehabilitation Science, Philadelphia, PA 19107, USA; 3 Neurodelphus LLC., Merion Station, PA 19066, USA

**Keywords:** essential tremor, wearable technology, rehabilitation engineering, occupational therapy, assistive technology, compression garment, inertial measurement unit, handwriting, feasibility study

## Abstract

Essential tremor (ET) often remains disabling despite pharmacologic, surgical, and stimulation-based options. This feasibility study evaluated LaceUp, a passive wearable sleeve combining soft compression with distributed embedded weight. Ten adults with ET completed a single laboratory visit with a fixed task battery under baseline, unweighted compression sleeve, wrist weight, and LaceUp conditions. Outcomes included wrist inertial measurement unit (IMU) tremor-band power (4–12 Hz), root mean square (RMS) jerk, digitized Archimedes spirals, handwriting, satisfaction/preference surveys, and follow-up Canadian Occupational Performance Measure (COPM) ratings. On the more-affected limb, LaceUp was associated with the largest median tremor-band power reduction (−32.9%) versus wrist weights (−25.3%) and the unweighted sleeve (−19.5%), while RMS jerk reductions were similar across conditions. Spiral and handwriting measures were heterogeneous and appeared most informative in participants with greater baseline severity. Six of nine participants who completed the preference ranking selected LaceUp as preferred. At follow-up, two of six reported continued use, and one exceeded the commonly cited 2-point COPM satisfaction threshold. Because this small pilot used a fixed order, effects cannot be separated from period, learning, expectancy, fatigue, behavioral adaptation, or measurement effects. Findings support feasibility and identify candidate endpoints, severity-based enrollment considerations, and operational constraints for future studies.

## 1. Introduction

### 1.1. The Burden of Essential Tremor

Essential tremor (ET) is the most common adult movement disorder, and its prevalence rises sharply with age; in one community-based U.S. multi-ethnic sample, prevalence reached 5.5% among adults older than 65 years [1]. ET is characterized by a 4–12 Hz upper-limb action tremor that emerges with posture and worsens during goal-directed movement, disrupting daily activities such as eating, drinking, writing, dressing, grooming, fastening buttons, and using a phone or keyboard. Functional impact is common, with one recent study reporting activities of daily living (ADL) impairment in 73% of patients and disability in 62%, particularly for eating, drinking, and writing [2]. These functional consequences motivated the present focus on task-based outcomes rather than tremor amplitude alone.

### 1.2. Distinguishing Essential Tremor from Physiologic Tremor

ET should be distinguished from physiologic or enhanced physiologic tremor. Physiologic tremor is a low-amplitude oscillation present in healthy individuals, whereas enhanced physiologic tremor reflects amplification by factors such as stress, caffeine, beta-agonists, hyperthyroidism, or fatigue [3,4]. ET, by contrast, is associated with abnormal oscillatory activity in cerebello–thalamo–cortical networks and typically presents as a persistent 4–12 Hz action tremor [5]. This distinction matters because distal mass loading can dampen mechanically driven oscillation, but in ET, it may instead alter mechanical-reflex resonance relative to the central drive frequency [6].

### 1.3. Available Interventions

First-line pharmacotherapy with propranolol or primidone can reduce tremor amplitude, but response is variable, and tolerability can be limited [7,8]. Surgical and stimulation-based options, including deep brain stimulation (DBS), focused ultrasound, and wrist-worn transcutaneous afferent patterned stimulation (TAPS), can provide greater tremor reduction for selected patients, but they introduce procedural, adverse-effect, calibration, adherence, or access constraints [9,10,11,12,13,14]. A substantial group therefore remains underserved: patients with mild-to-moderate disability who decline or are ineligible for surgery, who experience or fear medication side effects, or who want an intervention that can be donned for a specific task. Lightweight, passive, reusable garments may help fill this gap.

Mechanical approaches rely on the principle that distal mass can dampen high-frequency oscillation and have shown proof of concept in orthoses and exoskeletons, although many devices remain rigid, conspicuous, powered, or costly [6,15,16,17,18]. Compression garments may also alter sensory feedback and improve upper-limb movement precision [19,20]. The LaceUp sleeve combines circumferential forearm compression with distributed embedded mass. We therefore conducted a within-subject pilot to test whether this sleeve was associated with detectable reductions in objectively measured tremor relative to its component controls, an unweighted sleeve and a generic wrist weight, during a single visit, with follow-up to assess acceptability and continued use.

## 2. Materials and Methods

### 2.1. Design and Participants

We conducted an Institutional Review Board (IRB)-approved mixed-methods study at Thomas Jefferson University (iRISID-2025-0743, lab phase; iRISID-2026-0067, follow-up phase). The study included a single-visit, within-subject, fixed-order feasibility pilot and a follow-up qualitative interview. Participants were adults with essential tremor confirmed by a movement-disorders neurologist (J.R., T.W.L.). Ten participants were enrolled (five females, five males; ages 54–80 years, median 73 years; mean time since diagnosis 7.1 years, range 1–15 years). P010 (male participant) IMU data were unavailable because of an IMU file-generation error; therefore, the analyzable IMU cohort comprised five females and four males. The follow-up interview cohort comprised five males and one female. All were non-Hispanic and self-identified as White. Participants were recruited by convenience and snowball sampling from Jefferson neurology clinics. Inclusion criteria were age ≥ 18 years, clinical diagnosis of ET, sufficient cognitive capacity for informed consent and task performance, and forearm length/size compatible with available sleeve sizes. Exclusion criteria were pregnancy, active dermatologic disease at the sleeve site, uncontrolled epilepsy, and inability to complete a 120 min session. Nine participants yielded analyzable bilateral IMU recordings (P001–P009). All ten yielded spiral data from both pen-on-paper drawing and finger tracing on a digital tablet. Handwriting legibility data were available for nine participants (not collected for P008), and time-to-complete data were available for eight. Six participants completed the follow-up interview (P002, P003, P006, P007, P009, and P010; five males, one female; median time since the in-person LaceUp visit, 5 weeks; range, 2–16 weeks).

### 2.2. Device Description

The LaceUp device (LaceUp Athletics, Malvern, PA, USA) is a forearm-worn compressive elastic sleeve with an adjustable lace-up closure and integrated removable flexible (FLX) weights. The sleeve body is made from LYCRA compression material (The LYCRA Company, Wilmington, DE, USA) and is available in two sizes: Small/Medium for forearms measuring 8–12 inches from wrist to elbow and Large for forearms measuring 13–15 inches. According to manufacturer-provided specifications, the FLX Weight inserts are fabricated from a proprietary flexible composite in which atomized high-iron particles are dispersed within a polymer matrix. The material is injection moldable, allowing anatomically conforming flexible inserts to be fabricated with consistent geometry and mass distribution. The manufacturer also reports that the inserts are formulated using non-toxic, food-grade base materials and are engineered to have a skin-like durometer, allowing them to flex and conform to the forearm during functional movement. This compliant construction is intended to preserve mobility, reduce localized pressure points, reduce interference with natural joint motion, and avoid the contact hazards associated with rigid metal weights during physical contact or accidental impact.

Together, the flexible composite weights and Lycra compression provide a low-profile wearable configuration intended to deliver distributed external loading while limiting restriction of forearm and wrist movement. These properties may be relevant for tremor-related functional tasks, although their clinical contribution requires empirical evaluation. In the present study, the sleeve provided adjustable compression through the lace-up closure, but compression pressure was not instrumented or quantitatively measured.

### 2.3. Conditions and Sequence

**Figure 1:**
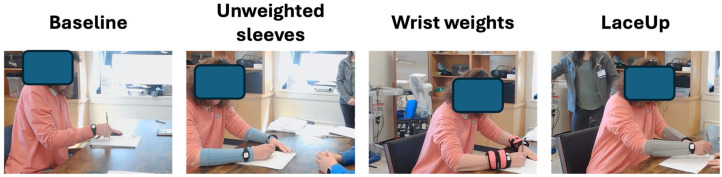
Representative photographs of a participant performing the study activities under the four experimental conditions: baseline without device, unweighted compression sleeve, commercial wrist weights, and LaceUp compression–weight sleeve. The images illustrate the fixed within-session condition sequence and the supervised task context used during laboratory testing.

The LaceUp device was evaluated within a fixed four-condition laboratory sequence (Figure 1). Each participant completed the task battery under four conditions: (i) baseline, no device; (ii) unweighted sleeve, a compression sleeve matched for material and appearance (sham/component control); (iii) wrist weights, a commercially available cuff weight matched for added mass without compression; and (iv) LaceUp, the experimental compression–weight sleeve. Conditions were administered in a fixed, step-up sequence rather than a randomized crossover design [21] because of fatigue and session-duration constraints. Wireless IMUs required multi-minute data transfers between conditions, and a standard four-condition session already approached the fatigue threshold for older participants with action tremor. Randomization would also have required longer washout periods to limit carryover, which was not feasible in this single-visit pilot and likely would have reduced data quality through participant exhaustion. The fixed sequence therefore progressed from the least to the most manipulative condition to establish an uncontaminated baseline and isolate component effects before the full intervention. Sensitivity observations addressing the time-on-task confound are presented in Section 3.9, and implications for future studies are discussed in Section 4.3. Sensor and stylus data were processed offline using coded condition files. Analysts were not blinded to condition identity, but analyses used prespecified processing scripts.

In the present study, total added mass was standardized at 1.5 pounds (0.68 kg), irrespective of sleeve size, with mass distributed uniformly along the length of the sleeve using four removable FLX Weight inserts positioned circumferentially. Each insert measured 7.5 inches in length and 1 inch in width and weighed 0.375 pounds, producing distributed forearm loading rather than focal distal loading at the wrist. Sleeves were selected according to forearm length and fitted by study personnel using a standardized approach: the LaceUp was progressively slid up the arm until the sleeve was secure, non-slipping during movement, and in consistent contact with the forearm without restricting circulation or active hand and wrist motion. No additional customization was performed.

For the wrist-weight condition, commercially available wrist weights totaling 1.5 pounds were applied at the wrist. These weights were selected to match the total added mass of the LaceUp sleeve but, by design, produced distal wrist-focused loading rather than distributed forearm loading. No additional matching beyond total mass equivalence was performed, and this difference in weight distribution was intentional. Participants donned the assigned device under supervision at the beginning of each experimental condition. The device was worn continuously for the full task block for that condition and removed before the subsequent condition. No intermittent removal or adjustment occurred during any condition unless required for safety; this did not arise during testing.

### 2.4. Outcome Measures

Inertial measurement units (IMUs). Wireless wrist-mounted IMUs (MetaMotionS; MbientLab Inc., San Jose, CA, USA) continuously recorded linear gravity-compensated acceleration during a fixed task block (across conditions and participants): postural hold, kinetic reach-and-touch, and writing/spiral. MetaMotionS is a wearable 10-axis sensing platform that includes a 3-axis accelerometer, 3-axis gyroscope, 3-axis magnetometer, and barometric sensor, with Bluetooth Low Energy 5.0 communication, USB data download, and 512 MB onboard flash memory for data logging; the device is ultra-lightweight (approximately 0.2 oz). For clarity, MbientLab product materials describe the encased MetaMotionS device as 27 mm × 27 mm × 4 mm, whereas MbientLab technical documentation lists the internal board dimensions as 17 mm × 25 mm × 5 mm; accelerometer and gyroscope sampling are supported up to 800 Hz in logging mode [22]. From the gravity-compensated acceleration data (collected at 100 Hz), two metrics were extracted offline using Python 3.13: (a) tremor-band power, the integral of the power spectral density of resultant acceleration in the 4–12 Hz band capturing pathological oscillation, and (b) RMS jerk, the root-mean-square of the time derivative of three-dimensional acceleration, a model-free index of movement smoothness.

For IMU analysis, left- and right-sided accelerometer files were processed separately for each participant and condition and then summarized at the participant–side–condition level. Raw timestamps were sorted, and the sampling frequency was estimated from the median positive inter-sample interval (nominal 100 Hz). Resultant acceleration magnitude was calculated as the Euclidean norm of the three acceleration axes:

$$a=\sqrt{a_{x}^{2}+a_{y}^{2}+a_{z}^{2}}$$

Recordings were segmented into overlapping 4 s windows with a 1 s step. Within each window, we computed RMS acceleration magnitude, tremor-band power, and RMS jerk. Tremor-band power was estimated from resultant acceleration using Welch power spectral density and integrated over 4–12 Hz, the canonical essential tremor band. For jerk analysis, each acceleration axis was low-pass filtered at 15 Hz with zero-phase filtering, differentiated with respect to time, and combined as the root-sum-square jerk magnitude. Primary analyses were restricted to active windows, defined for each participant, side, and condition as windows with RMS acceleration at or above the median + 0.5 × interquartile range. This threshold was chosen as a simple, robust, distribution-based rule to reduce the influence of pauses, transitions, and very low-movement windows within the fixed task block while avoiding manual annotation of task epochs. Using the median and interquartile range made the threshold less sensitive to brief high-amplitude artifacts than a mean/standard-deviation rule, and applying it separately within each participant–side–condition recording allowed the active-window definition to adapt to differences in baseline movement amplitude across participants and limbs. Condition-level IMU summaries were the median active-window tremor-band power and median active-window RMS jerk. The primary descriptive IMU endpoint was percent change from baseline in median active-window 4–12 Hz tremor-band power on the more-affected limb, defined a priori as the limb with higher baseline tremor-band power. Because this definition uses the same baseline recording as the response calculation, the endpoint is vulnerable to regression to the mean and is interpreted descriptively. For transparency, participant-level raw baseline values and absolute changes from baseline are reported in addition to ratio-based summaries (Supplementary Table S1). For each participant, side, metric, and intervention condition, effects were normalized to the corresponding baseline value and expressed as intervention-to-baseline ratios and percent change. Ratios < 1 and negative percent changes indicate reduction relative to baseline. In addition to left- and right-side analyses, we report summaries by within-participant lateralization, defined as the more-affected versus less-affected limb. We also report an exploratory overall response defined as the lower (better) of the two side-specific ratios. Because this definition selects the better response within each participant, it is inherently optimistic and was not treated as the principal IMU result. Baseline tremor severity was defined as the maximum 4–12 Hz tremor-band power observed across limbs during baseline. Participants were ranked by this value and divided into low, moderate, and high tertiles (*n* = 3 per tertile).

Digitized Archimedes spiral. Participants completed The Essential Tremor Rating Assessment Scale (TETRAS) spiral on a stylus-enabled tablet running the open-source TRSPER application [23], which exports per-trial geometric and kinematic descriptors: chance-line crossings, first- and second-degree smoothness, first- and second-degree zero crossings, mean Δr (radial deviation), and Δr/time (defined in Table 1). Two trials per condition per hand were attempted.

Handwriting Assessment Battery for Adults (HAB). Participants completed the Writing Legibility subtest of the HAB version 6, for which they wrote a five-word sentence of their choosing in their preferred style of writing. They repeated the same sentence for each condition. An occupational therapist (OT) or OT doctoral capstone student scored a global legibility rating and a per-word legibility rating on a 1 (illegible) to 4 (legible) ordinal scale, plus time-to-complete in seconds. The HAB has demonstrated high inter-rater reliability for legibility subtests (ICC 0.71–0.83), with a documented ceiling effect on shorter or simpler writing samples [24]; no published minimum detectable change (MDC) or minimum clinically important difference (MCID) is currently available for HAB-v6 legibility scores.

Patient-reported, feasibility, and qualitative measures. Feasibility outcomes were defined as laboratory tolerability, task completion, data completeness, acceptability, and exploratory continued use. Participants completed the WHO-5 Well-being Index, the TETRAS-ADL questionnaire [25], a per-condition satisfaction rating (1–5 ordinal), a four-condition preference ranking, and the Canadian Occupational Performance Measure (COPM; administered from P006 onward, excluding P008). The COPM elicits each participant’s five most important performance problems and rates performance and satisfaction on 0–10 scales, with higher scores indicating greater performance and satisfaction; the published MCID is a 2-point change [26]. Six of the ten lab-phase participants (P002, P003, P006, P007, P009, P010) completed a structured follow-up telephone interview, a median of 5 weeks post-visit, using a hybrid deductive/inductive coding framework and NVivo 15.5.1 software (Lumivero, LLC., Denver, CO, USA). Two independent OT doctoral capstone student coders performed the analysis.

### 2.5. Statistical Approach

All quantitative comparisons were within participants and referenced to baseline. Continuous sensor outcomes (IMU tremor-band power and RMS jerk) were summarized as intervention-to-baseline ratios and percent change for each participant and limb. Group summaries are reported primarily as medians and interquartile ranges because of the small sample and expected non-normality. The primary descriptive IMU endpoint was percent change from baseline in 4–12 Hz tremor-band power on the more-affected limb, defined a priori as the limb with higher baseline tremor-band power. Analyses are presented by limb (left, right) and by within-participant lateralization. We also report an exploratory overall response defined as the lower (better) of the two limb-specific ratios, capturing improvement on at least one limb. Because this summary selects the better of two responses within each participant, it is expected to be optimistic and is not emphasized as a principal result.

Because this feasibility pilot used a fixed condition order, statistical testing was exploratory and hypothesis-generating rather than confirmatory. For each IMU metric and side, we performed paired nonparametric Wilcoxon signed-rank tests comparing each intervention condition (unweighted sleeve, wrist weights, LaceUp) against baseline, with Holm correction across the three comparisons within each metric-side stratum. Complete exploratory results, including n, median ratio, median percent change, Wilcoxon signed-rank statistic, unadjusted *p*-value, and Holm-adjusted *p*-value, are reported in Supplementary Table S2. These tests were not used to support confirmatory efficacy claims. Participants were classified as responders when the intervention-to-baseline ratio was <1.0. To describe heterogeneity, we summarize outcomes on the more-affected versus less-affected limb and by descriptive severity strata (*n* = 3 per tertile), defined by maximum baseline tremor-band power across limbs. These strata are not interpreted as evidence of severity-modulated efficacy because cell sizes are small and the same metric is used to define severity and compute response, introducing regression-to-the-mean bias. Ratio-based outcomes are unstable when baseline values are small [27,28], especially on the less-affected limb. Digitized spiral and HAB legibility outcomes were ordinal or non-normally distributed and were therefore summarized descriptively, without multiplicity-adjusted inferential claims. HAB time-to-complete is reported at the participant level across all four conditions to assess time-on-task effects (Section 3.9). Satisfaction ratings, preference rankings, and follow-up COPM changes are summarized descriptively.

## 3. Results

### 3.1. Participants

Nine participants (P001–P009) contributed bilateral IMU data (median age 73 years, range 54–80 years; five females, four males); all ten contributed spiral data. Handwriting legibility data were available for nine participants, with time-to-complete data for eight; participant-level data availability across laboratory and follow-up measures is summarized in Supplementary Table S3. Seven were right-handed, one was left-handed (P008), and two reported ambidextrous use (P005, P009). Tremor duration ranged from a few years to several decades, with substantial inter-limb asymmetry on baseline IMU recording: the ratio of more-affected to less-affected baseline tremor-band power exceeded 2:1 in five of nine participants. Overall baseline severity, indexed by the maximum baseline tremor-band power observed across limbs, spanned approximately 13.5-fold across the cohort.

### 3.2. Aggregate Effects Across Conditions

**Figure 2:**
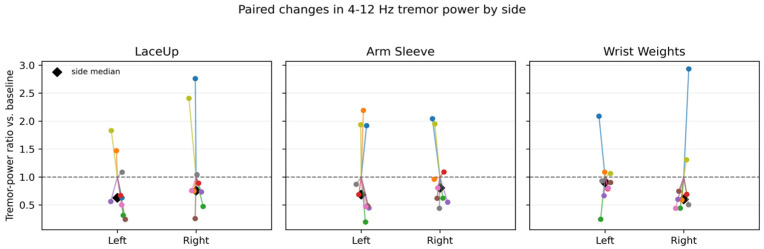
Limb-specific baseline-to-intervention changes in 4–12 Hz tremor-band power by condition. Colors distinguish participants; each colored line represents one participant-limb comparison from the baseline ratio of 1.0 to the intervention ratio; black diamonds indicate limb-specific medians. Ratios below 1.0 indicate lower tremor-band power relative to baseline. These limb-specific summaries are descriptive, are not the primary descriptive IMU endpoint, and are distinct from the exploratory overall-response metric shown in Figure 5.

Across participant-level medians collapsed across limbs, all three interventions were associated with lower tremor-band power and RMS jerk relative to baseline (Figure 2 and Figure 5). Complete exploratory Wilcoxon signed-rank results with Holm-adjusted *p*-values are provided in Supplementary Table S2 and should be interpreted only as hypothesis-generating because of the small sample size and fixed condition order. Aggregate medians did not clearly distinguish the three conditions: tremor-band power reductions were −34.5% for the unweighted sleeve, −26.0% for wrist weights, and −21.9% for LaceUp; RMS jerk reductions were similar (LaceUp −20.2%, sleeve −20.1%, weights −18.4%). These limb-based medians differ from the Figure 5 exploratory overall-response values because Figure 5 defines overall response as the better of the two limb-specific responses for each participant. This summary is inherently optimistic and is shown only to indicate whether any limb improved within a participant, not as the principal IMU outcome. Using this at-least-one-limb responder definition, seven of nine participants (77.8%) responded to LaceUp, the same rate observed for the sleeve and wrist weights. The two non-responders to LaceUp (P008, P009) had the lowest baseline tremor-band power in the cohort; P009 worsened under all three conditions, suggesting possible measurement-floor or low-baseline variability effects.

### 3.3. Lateralization: The More-Affected Limb Carries the Signal

**Figure 3:**
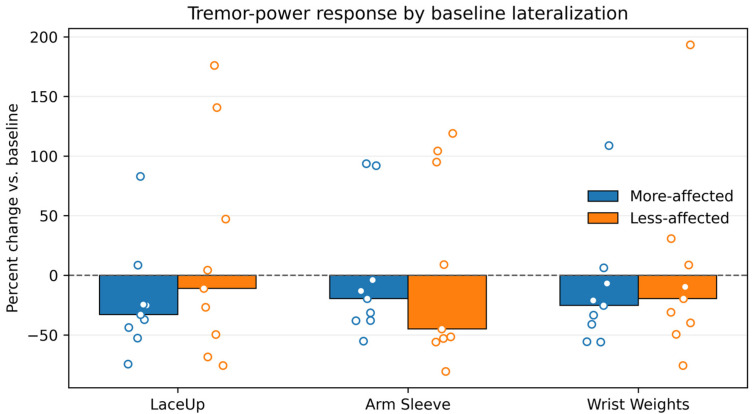
Primary descriptive IMU endpoint: percent change in 4–12 Hz tremor-band power on the more-affected and less-affected limbs. Bars show group medians, and open circles show individual participant-limb values. Negative values indicate lower tremor-band power relative to baseline. The more-affected limb was defined by the larger baseline tremor-band power, so this endpoint should be interpreted descriptively and with awareness of regression-to-the-mean risk.

When results were stratified by within-participant lateralization, the effects were concentrated on the more-affected limb. Median tremor-band power reduction was −32.9% for LaceUp, −25.3% for wrist weights, and −19.5% for the unweighted sleeve (Table 2). Tremor-band power values are integrated 4–12 Hz acceleration power from wrist IMU acceleration, in (m/s^2^)^2^. Supplementary Table S1 provides participant-level bilateral baseline values, baseline asymmetry ratios, and condition values with absolute changes for the baseline-defined more-affected limb. On the less-affected limb, LaceUp showed a median reduction of −10.9% (mean +15.2%); the unweighted sleeve showed a larger median reduction (−45.0%) but a positive mean (+4.6%). These less-affected-limb summaries likely reflect instability of ratio-based outcomes when baseline values are near the measurement floor, where small absolute changes can produce large ratios. Overall, the more-affected limb provided the most interpretable descriptive pattern (Figure 3).

### 3.4. Severity Stratification

**Figure 4:**
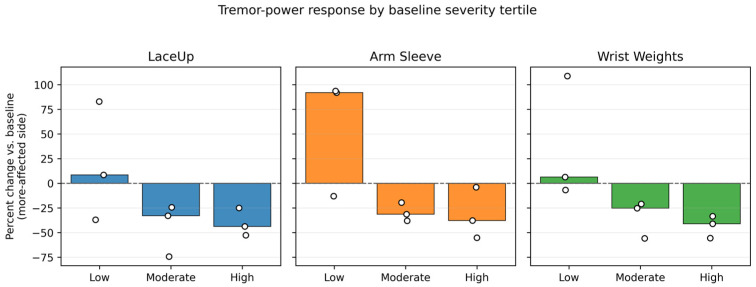
Severity-stratified percent change in 4–12 Hz tremor-band power on the more-affected limb. Participants were divided into low, moderate, and high tertiles (*n* = 3 each) using the maximum baseline tremor-band power observed across limbs. Bars show median percent change relative to baseline, and open circles show individual participant values. These strata are descriptive because baseline tremor power was used both to define severity and to calculate response ratios. They are intended to motivate future enrollment and stratification criteria, not to support efficacy inference.

Tertiles based on maximum baseline tremor-band power across limbs are summarized in Figure 4. In the high-severity tertile (*n* = 3), median reductions occurred under all three conditions (LaceUp −43.8%, wrist weights −41.0%, sleeve −37.8%). In the moderate-severity tertile (*n* = 3), LaceUp showed the largest numerical reduction (−32.9%), followed by the sleeve (−31.4%) and weights (−25.3%). In the low-severity tertile (*n* = 3), median worsening occurred under all three conditions (sleeve +91.9%, LaceUp +8.5%, weights +6.3%). With only three participants per cell, these estimates are descriptive and cannot support inference about severity-modulated efficacy. Two caveats should be noted. First, the same metric is used to define severity strata and compute response ratios, introducing regression-to-the-mean bias. Second, the low-severity tertile is closest to the measurement floor, where ratio-based outcomes are unstable. We therefore present this tertile pattern as a descriptive observation to motivate severity-based enrollment in future work (Section 4.3), not as evidence of differential efficacy.

### 3.5. Cross-Metric Consistency: Tremor-Band Power and RMS Jerk

**Figure 5:**
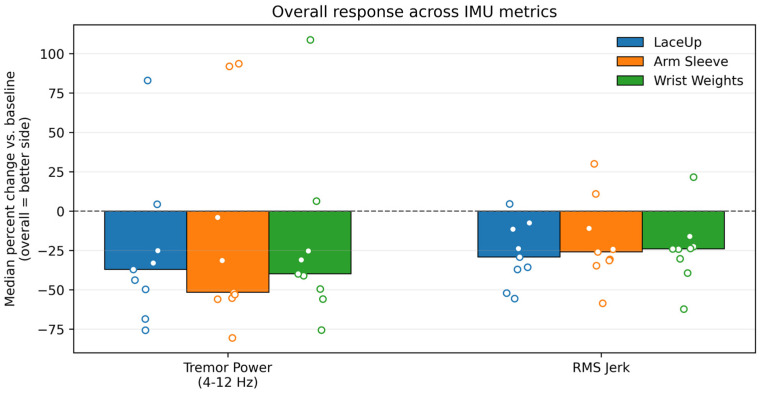
Exploratory overall IMU response across tremor-related metrics. Bars represent the median percent change relative to baseline for 4–12 Hz tremor-band power and RMS jerk, using an overall participant-level response defined as the better of the two limb-specific responses. Open circles show individual participant values. Because this metric selects the better of two limb-specific responses within each participant, it is inherently optimistic and was not treated as the principal IMU result.

Using the overall-response definition shown in Figure 5, RMS jerk showed median reductions of −29.2% for LaceUp, −25.9% for the unweighted sleeve, and −24.0% for wrist weights. Agreement between an oscillation-specific spectral measure (4–12 Hz tremor-band power) and a model-free smoothness measure (jerk) suggests that both metrics captured changes in movement quality during the task. This partly reduces concern that lower tremor-band power only reflects mass loading shifting the limb’s resonant frequency outside the 4–12 Hz analysis window. However, RMS jerk is broadband and can also change with voluntary movement strategy, co-contraction, attentional slowing, and altered reach kinematics. Parallel reductions in jerk and tremor-band power therefore make a real movement change more plausible but do not prove specific suppression of pathological oscillation. That distinction will require mechanistic studies with calibrated absolute-scale endpoints and per-condition baseline remeasurement.

### 3.6. Exemplar Individual Responses (Handwriting and Spiral Samples)

**Figure 6:**
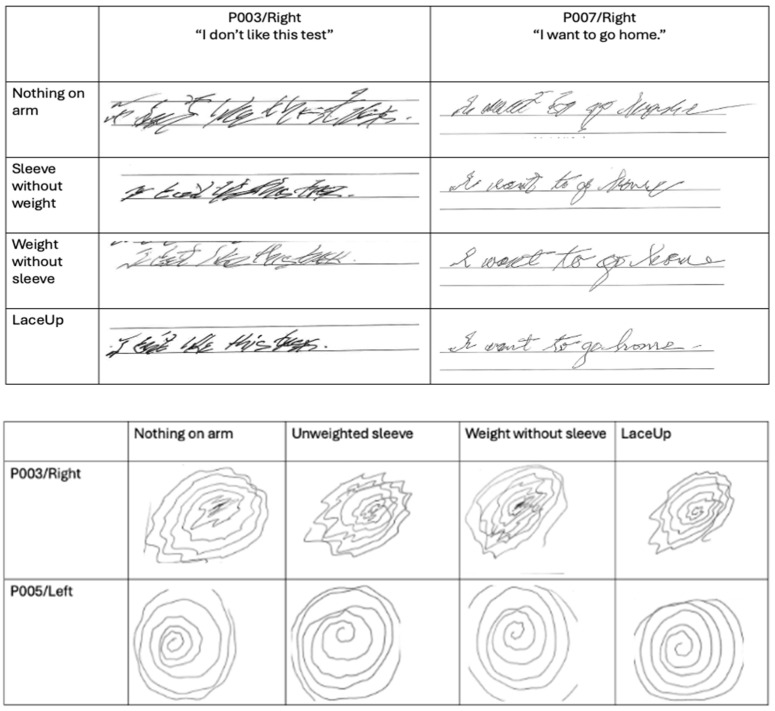
Representative handwriting samples (**top image**) and spiral drawings (**bottom image**) from selected participants across study conditions. Examples are shown in administered order, comparing baseline, unweighted sleeve, wrist weights, and LaceUp. The figure illustrates qualitative variation in spiral quality and handwritten output, including visually apparent change, minimal change, and between-participant heterogeneity. These examples are illustrative and are not used as evidence of efficacy.

To complement the cohort-level analyses with a qualitative impression of the within-session response, we present representative handwriting and spiral samples in Figure 6. These exemplars are illustrative, not evidentiary; the cohort-level data including non-responders are reported in Section 3.2, Section 3.3, Section 3.4, Section 3.5, Section 3.7 and Section 3.8.

### 3.7. Digitized Spiral Metrics

**Figure 7:**
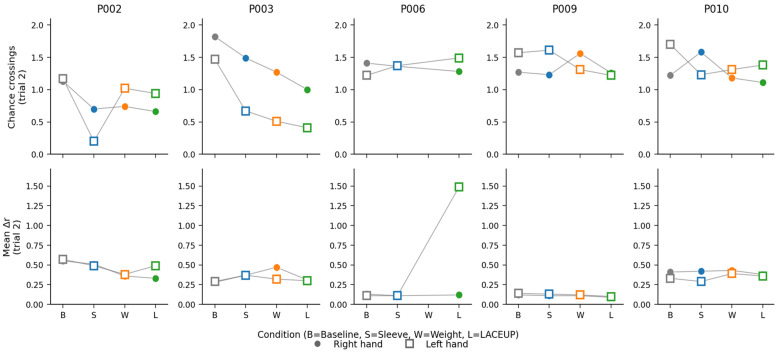
Selected digitized spiral metrics from TETRAS spiral drawings across conditions (trial 2), illustrating per-participant heterogeneity. Top row: chance-line crossings; bottom row: mean Δr (radial deviation). Colors distinguish conditions. Filled circles = right hand; open squares = left hand. Conditions are plotted in administered order (B = baseline, S = unweighted sleeve, W = wrist weights, L = LaceUp). P002 and P003 show LaceUp-favoring trends; P006 and P010 show largely flat patterns; P009 shows minimal change. P006’s mean Δr of 1.49 on the left hand under LaceUp was confirmed to match the manually entered source record, but the original TRSPER export was not retrievable; it is therefore treated as an unresolved source-data anomaly and excluded from interpretive conclusions.

The digitized Archimedes spiral, captured with TRSPER, provided a bedside complement to IMU recording. Across the seven prespecified spiral descriptors (Table 1), patterns were heterogeneous and showed the same apparent severity dependence seen in the IMU data. P002, who had moderate baseline tremor, showed a marked LaceUp-specific reduction in chance-line crossings on both hands and the lowest mean Δr of any condition on the right hand. P003, the participant with the highest baseline severity, showed a monotonic LaceUp-favoring reduction in chance-line crossings on both hands (right: 1.82 → 1.00; left: 1.47 → 0.41 across the four sequential conditions). P006 and P010 showed essentially flat chance-line crossings across conditions, paralleling their flat IMU response. P010 was at the cohort floor of severity. P006 had high IMU severity, but spiral chance-line crossings were already near that participant’s floor before LaceUp. P009 showed minimal change in either metric across conditions on either hand, consistent with bilateral worsening in the IMU data and supporting a measurement-floor or low-baseline variability interpretation. Direction reversals across hands within the same participant under the same condition, most visible in P006 and P009 in Figure 7, argue against a pure time-on-task confound; this is discussed in Section 3.9. Trial 2 measurements showed enough within-session repeatability to support spiral metrics as a screening signal, but nontrivial test–retest variance indicates that confirmatory protocols should use duplicate trials.

### 3.8. Handwriting Legibility and Time

Occupational therapy clinician/student ratings of a five-word sentence showed a strong floor/ceiling pattern. Most global and per-word legibility ratings clustered at 3 or 4 of 4, and most participants showed no condition-related change. Even so, sample-mean global legibility increased slightly across conditions (baseline 3.1 ± 1.3, sleeve 3.1 ± 1.1, weights 3.1 ± 1.1, LaceUp 3.3 ± 0.9), with the highest mean under LaceUp. Two participants (P003 and P007), both with high tremor severity, improved by ≥1 ordinal point under LaceUp relative to baseline; for P007, this pattern was consistent across all five rated words.

**Figure 8:**
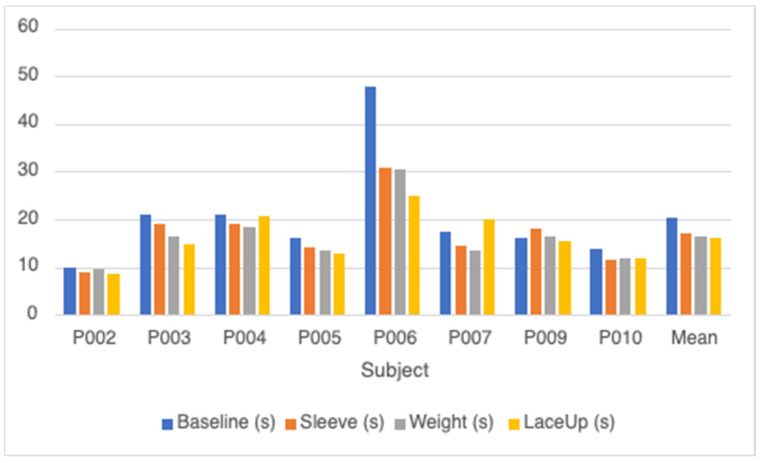
HAB time-to-complete (seconds) for the fixed five-word sentence by participant and condition, administered in fixed order (Baseline, Unweighted sleeve, Wrist weights, LaceUp). Lower values indicate faster completion. P001 is not shown because HAB time-to-complete was not collected; P008 is not shown because HAB was not completed.

Time-to-complete differed substantially across conditions, even among participants whose ordinal legibility ratings were at ceiling, with the fastest writing times often observed under LaceUp (Figure 8). Across the eight participants with time data, mean writing time was 20.3 s at baseline (SD = 11.7), 17.0 s under the unweighted sleeve (SD = 6.6), 16.3 s under wrist weights (SD = 6.4), and 16.1 s under LaceUp (SD = 5.3) (Table 3). Several participants showed marked LaceUp-associated reductions: P006 wrote the same sentence in 47.9 s at baseline, 30.6 s under wrist weights, and 24.9 s under LaceUp (48% reduction from baseline) while remaining at the same ordinal legibility tier. P003 decreased from 20.9 s at baseline to 14.8 s under LaceUp, with a 1-point increase in legibility. Conversely, P004 and P007 showed nonmonotonic patterns in which performance worsened at the LaceUp step (P007: 13.5 s under wrist weights to 20.1 s under LaceUp), confirming heterogeneous within-session response. These findings suggest that, when ordinal ratings are at ceiling, time-to-complete should be reported alongside legibility because it may preserve within-session signal.

### 3.9. Sensitivity Analyses Against a Pure Time-on-Task Confound

Because conditions were administered in a fixed sequence, we examined whether the observed patterns could be explained by a monotonic time-on-task effect. Several descriptive observations were not fully consistent with that explanation: individual HAB time-to-complete trajectories were nonmonotonic in several participants, spiral metrics showed directionally different responses across limbs within the same participant, and the strongest IMU tremor-power signal was concentrated on the more-affected limb rather than appearing uniformly across both limbs. These observations do not rule out period, learning, fatigue, expectancy, behavioral adaptation, or measurement effects; they only suggest that a simple linear time trend may not fully explain the findings.

We did not perform a formal mixed-effects detrending analysis on the IMU data because the current per-condition extractions do not retain the intra-condition timestamps needed to model time-within-condition as a covariate. Accordingly, the dataset cannot fully separate condition effects from within-session temporal trends. This limitation informs interpretation of the findings and future protocol development, where direct detrending and time-resolved modeling would be appropriate.

### 3.10. Patient-Reported Satisfaction and Condition Preference

After each condition, participants rated satisfaction with their performance on a 1–5 ordinal scale. Mean satisfaction increased monotonically across conditions: baseline 3.3 ± 1.6, unweighted sleeve 3.4 ± 1.4, wrist weights 3.6 ± 1.1, LaceUp 3.8 ± 1.3 (Table 4). At the end of the session, participants ranked the four conditions by preference. Six of nine participants who completed the ranking (66.7%) selected LaceUp as most preferred, two selected baseline, one selected the unweighted sleeve, and none selected wrist weights alone (Table 5). Reasons for preferring LaceUp centered on perceived steadiness, improved water-pour performance, and greater comfort or less bulk than wrist weights. Representative comments included: “more steady with weighted sleeve, more confidence in water pour with less spill,” “the weighted sleeve felt good and was a bit more effective than the weight, felt better and less bulky,” and “prefer the weight on one side versus the two.”

No adverse events, skin injury, circulatory compromise, or transient discomfort were reported or observed during laboratory testing. Participants were explicitly asked about discomfort during and after each condition, and none was recorded. No condition required device removal or adjustment for safety. The later report of discomfort during unsupervised home use occurred after the laboratory session and is described separately in the follow-up findings.

### 3.11. Discarded Metric: 9-Hole Peg Test (9HPT)

We initially collected 9HPT performance for the first five participants. Under non-baseline conditions, some participants moved more slowly and carefully to preserve accuracy, increasing completion time. Because the 9HPT measured only time to complete and did not capture movement accuracy, we concluded that it was not informative in this context and discontinued its use.

### 3.12. Follow-Up: Exploratory Post-Study Use and Qualitative Themes

Six of the ten lab-phase participants completed a follow-up telephone interview at a median of 5 weeks post-visit (range 2–16 weeks). Post-study device use was not a prespecified outcome and was not systematically assessed. Sleeves were provided without standardized instructions, training, adherence monitoring, or scheduled follow-up support; therefore, subsequent use or non-use reflects unstructured, unsupervised behavior rather than adherence to a defined intervention. Two of six interviewees reported continued everyday LaceUp use: one wore the weighted sleeve bilaterally during all waking hours for several months and reported “somewhat improved” arm function; the other wore the sleeves daily for approximately 30 min during meals. Both rated themselves “highly likely” to recommend LaceUp to other ET patients. Four reported no continued use. Two described the sleeves as too heavy for sustained functional wear despite finding them comfortable during the initial trial, and one reported temperature-related discomfort. The observed pattern should therefore not be over-interpreted as evidence of real-world effectiveness.

Four of the six interviewees completed the COPM at both time points; P002 and P003 completed follow-up interviews but were enrolled before COPM administration began. The participant who continued daily LaceUp use during meals showed a +3.8 increase in mean COPM satisfaction, above the commonly cited 2-point threshold, with a +6 increase in satisfaction for self-feeding and a +3 increase in self-feeding performance. The other three follow-up COPM scores declined modestly or were essentially unchanged, consistent with reported non-use.

Hybrid deductive/inductive coding of interview transcripts identified five themes. First, tremor strongly affected occupational performance, especially eating, drinking, handwriting, and hobby-specific activities (representative quote: “I’m very interested in playing classical guitar…it really messes with my ability to do that”). Second, perceived sleeve benefit was polarized: some participants reported clear improvement (“a smaller range of movement with the shaking”), whereas others reported no meaningful effect. Third, desired outcomes centered on steadiness and precision rather than amplitude reduction alone. Fourth, tolerance concerns included size, weight, comfort, temperature, and aesthetics, with weight the most consistent reason for discontinuation (“no, they weren’t uncomfortable, they were just so heavy”). Fifth, several participants were exploring alternative treatments, including botulinum toxin or medication changes. These qualitative themes aligned with the laboratory feasibility findings, but they cannot disambiguate whether non-use reflected limited utility, inconvenience, or contextual factors unrelated to device performance or whether continued voluntary use by a subset reflected perceived benefit in appropriately selected individuals. Both interpretations are plausible, and the follow-up data collected here are insufficient to adjudicate between them.

## 4. Discussion

### 4.1. Principal Findings

This single-visit, fixed-order feasibility pilot supports a limited conclusion: LaceUp can be worn during a structured task battery, participants can complete the laboratory procedures, and the protocol can generate interpretable sensor, handwriting, spiral, acceptability, and follow-up data in a small cohort of adults with essential tremor. The prespecified feasibility outcomes were laboratory tolerability, task completion, data completeness, acceptability, and exploratory continued use; this study was not designed to evaluate real-world adoption, adherence, standardized home use, or effectiveness. The clearest quantitative signal was IMU tremor-band power on the more-affected limb, where LaceUp was associated with a median reduction of −32.9% from baseline compared with −25.3% for wrist weights and −19.5% for the unweighted sleeve. Participant-collapsed bilateral summaries were less discriminating: tremor-band power reductions were −34.5% for the unweighted sleeve, −26.0% for wrist weights, and −21.9% for LaceUp, while RMS jerk reductions were similar across conditions. Given the small sample and fixed order, these findings are best interpreted as descriptive feasibility signals rather than evidence of device superiority.

The more-affected-limb analysis appears more clinically interpretable than bilateral aggregation in this dataset. Baseline tremor was asymmetric, and ratio-based outcomes became unstable when baseline tremor power was low, especially in the low-severity tertile. Future studies should consider prespecifying a more-affected-limb endpoint and using stable-trait baseline severity, rather than same-day response to weight, when defining eligibility or stratification.

### 4.2. Outcome Selection

The pilot also clarified which outcomes were most informative. IMU tremor-band power was sensitive to within-session change, but its clinical interpretability remains limited without an external functional anchor. RMS jerk was a useful cross-check because it is a broadband measure of movement smoothness, although it cannot determine whether change reflects tremor suppression, altered movement strategy, co-contraction, or frequency shifting. Digitized spiral metrics and handwriting measures added face validity but were heterogeneous and showed floor or ceiling effects in some participants. Ordinal HAB legibility ratings were often near ceiling, whereas handwriting time-to-complete preserved useful within-session information. Although the HAB captures both speed and accuracy, the 9HPT had limited utility because it measured time without accuracy; other time-based fine motor assessments may share this limitation.

For future work, a defensible endpoint strategy would pair an objective sensor endpoint with a clinically interpretable task. Candidate primary endpoints include 4–12 Hz tremor-band power on the more-affected limb, a modified HAB time-to-complete using a sufficiently difficult writing sample, and a participant-selected ADL scored from video using a prespecified rubric, noting that blinding to condition is not feasible. A clinician-rated TETRAS performance subscale should be added to improve comparability with the essential tremor literature. Satisfaction, condition preference, exemplar handwriting or spiral samples, and follow-up interviews should remain secondary feasibility or acceptability outcomes.

### 4.3. Implications for Future Study Design

A practical next trial would test LaceUp against a prespecified comparator, most plausibly the unweighted sleeve to isolate the added effect of distributed mass, while retaining wrist weights as an active mechanical benchmark or secondary comparator. The primary analysis should be defined before enrollment, such as change in 4–12 Hz tremor-band power on the more-affected limb paired with a clinical or functional task rating. The protocol should also specify whether LaceUp is intended for unilateral use on the more-affected limb, bilateral use, or task-specific use because activities such as eating, writing, and drinking may require different wear patterns.

Three design features are especially important for a confirmatory study: randomized or counterbalanced condition order, repeated baseline assessments to estimate within-person variability, and enrollment or stratification criteria that account for baseline tremor severity. Together, these changes would move the work from feasibility and endpoint selection toward a direct test of reproducible, clinically interpretable benefit beyond sequence-related change.

The follow-up interviews suggest an additional device-development consideration. The weight that may contribute to short-term improvement was also a common reason for discontinuing home use. Real-world adoption and adherence therefore require prospective investigation with standardized instructions, structured follow-up, adherence monitoring, and predefined home-use endpoints. Future device iterations may need adjustable mass, task-specific versions, or guidance for occasion-specific wear, such as during meals or handwriting, rather than continuous daily use.

### 4.4. Limitations

The main limitations are the small sample, fixed condition order, and short observation period. The *n* = 9 to 10 cohort is too small for condition-specific inference, and tertile analyses contain only three participants per group. Because all participants completed conditions in the same sequence, intervention effects cannot be separated from period, learning, fatigue, expectancy, behavioral adaptation, or measurement effects. These structural limitations were partly unavoidable in this single-visit pilot and imply that all between-condition comparisons remain descriptive and exploratory. The unweighted sleeve matched material and appearance but did not blind participants to added mass. The cohort was demographically homogeneous and recruited from a single center, limiting generalizability. No clinician-rated TETRAS performance subscale was collected, limiting comparison with other essential tremor device studies.

Measurement limitations also matter. Ratio-based outcomes are unstable when baseline values are near the floor, particularly on the less-affected limb [27,28]. Spiral and handwriting outcomes showed participant-specific floor and ceiling effects [27]. Follow-up data were available for only six participants, with variable timing and no structured home-use protocol, so those findings are best treated as acceptability, tolerability, and device-refinement information [29,30]. Finally, P010 IMU data were unavailable because of an IMU file-generation error, and one P006 spiral value was retained only as an unresolved source-data anomaly after checking against the manually entered source record; neither issue was used to support interpretive claims.

### 4.5. Comparative Context

This pilot should not be compared numerically with DBS, focused ultrasound, or TAPS studies, even though the endpoints were equivalent. Those studies typically use clinician-rated tremor scales, whereas this pilot emphasized single-session IMU ratios and functional task measures. The relevant question for LaceUp is narrower: whether a passive, reusable garment can provide task-specific benefit for people who want a non-electronic option or who are not good candidates for medication, surgery, or stimulation-based devices.

## 5. Conclusions

This fixed-order feasibility pilot demonstrated that LaceUp can be tolerated during supervised laboratory testing and that the protocol reliably generates interpretable sensor, handwriting, spiral, acceptability, and follow-up data in adults with essential tremor. The most consistent quantitative signal was a substantial reduction in IMU tremor-band power on the more-affected limb under LaceUp (median −32.9% from baseline), which exceeded the reductions seen with either component control and converged with parallel reductions in RMS jerk and with favorable laboratory preference ratings. Because conditions were administered in a fixed order in a small cohort, this convergence cannot be attributed causally to the device or interpreted as superiority over the unweighted sleeve or wrist weights; rather, it establishes that the combined compression–weight configuration produces a measurable, multi-metric within-session signal worth testing under controlled conditions. Post-study use by a subset of follow-up interviewees provides acceptability and device-refinement information rather than evidence of real-world effectiveness. Collectively, this study identifies a feasible primary endpoint and supporting measures, demonstrates that baseline severity and limb asymmetry are decisive design considerations, and provides the operational basis for a randomized, counterbalanced trial powered to test whether LaceUp delivers reproducible, clinically meaningful benefit during tremor-relevant tasks.

## Figures and Tables

**Table 1:** Digitized Archimedes spiral metrics calculated by the open-source TRSPER application [23].

Metric	Definition	Range and Meaning
Chance-line crossings	Number of crossings relative to an expected baseline trajectory.	Lower = fewer irregular crossings and a smoother trace; higher = more irregular deviations.
1° smoothness	Log variance of first-order radial change (Δr/Δθ).	lower (more negative) = smoother trajectory; higher = irregular radial changes.
2° smoothness	Log variance of second-order change (curvature/acceleration of Δr/Δθ).	lower = stable curvature; higher = jerky curvature changes.
1° zero crossings	% of sign changes in first-order radial gradient.	lower = few oscillations; higher = frequent direction reversals.
2° zero crossings	% of sign changes in second-order term (curvature/acceleration).	lower = stable curvature; higher = rapid curvature fluctuations.
Mean Δr (radial deviation)	Mean incremental radial change between points (ri + 1 − ri).	More stable values = more controlled spiral growth; greater variability or excursions = less stable amplitude control.
Δr/time	Radial change normalized by time (radial velocity).	More stable values = more consistent movement speed; high variability or spikes = less consistent radial velocity.

**Table 2:** Summary of the primary descriptive IMU endpoint on the more-affected limb. Values are median percent change from baseline in 4–12 Hz tremor-band power; negative values indicate lower tremor-band power relative to baseline. Tremor-band power is integrated 4–12 Hz acceleration power from wrist IMU acceleration. Participant-level bilateral baseline values, baseline asymmetry ratios, and condition values with absolute changes for the baseline-defined more-affected limb are provided in Supplementary Table S1.

Condition	Median Percent Change from Baseline	Interpretation
Unweighted sleeve	−19.5%	Lower tremor-band power versus baseline
Wrist weights	−25.3%	Lower tremor-band power versus baseline
LaceUp	−32.9%	Largest descriptive median reduction

**Table 3:** HAB time-to-complete (seconds) by participant and condition. P006 shows a large LaceUp-specific reduction in writing time despite remaining at the same ordinal legibility tier, indicating that time-to-complete captures within-session signal that the ordinal rating misses. Δ LaceUp versus baseline is shown. In the mean row, the reported −14% is the mean of individual participant percent changes, not the percent change computed from the mean baseline time to the mean LaceUp time.

Subject	Baseline (s)	Unweighted Sleeve (s)	Wrist Weights (s)	LaceUp (s)	Δ LaceUp vs. Baseline
P002	9.78	9.06	9.65	8.69	−11%
P003	20.88	19.16	16.44	14.82	−29%
P004	20.94	19.13	18.40	20.53	−2%
P005	16.0	14.26	13.63	12.69	−21%
P006	47.90	30.72	30.63	24.87	−48%
P007	17.38	14.34	13.47	20.12	+16%
P009	15.97	18.03	16.44	15.50	−3%
P010	13.91	11.54	11.81	11.81	−15%
Mean	20.3	17.0	16.3	16.1	−14%

**Table 4:** Per-condition satisfaction ratings (1 = dissatisfied, 5 = satisfied), administered after each condition from participant 002 onward (*n* = 9).

Condition	Mean Satisfaction (1–5)	SD
Baseline	3.3	1.6
Unweighted sleeve	3.4	1.4
Wrist weights	3.6	1.1
LaceUp	3.8	1.3

**Table 5:** Condition preference ranking at the end of the session (*n* = 9). Participants ranked the four conditions; the most-preferred selection is shown.

Condition	Most-Preferred, *n* (%)
Baseline (no device)	2 (22%)
Unweighted sleeve	1 (11%)
Wrist weights	0 (0%)
LaceUp	6 (67%)

## Data Availability

The data generated and analyzed during the current study are not publicly available because they contain human-participant sensor, handwriting, and interview data collected under an IRB-approved feasibility protocol. De-identified IMU data, de-identified processed analysis tables, and the analysis scripts may be made available from the corresponding author upon reasonable request, subject to institutional and IRB restrictions.

## Supplementary Materials

The following supporting information can be downloaded at: https://www.mdpi.com/article/10.3390/bioengineering13070785/s1, Table S1: Participant-level bilateral baseline 4–12 Hz tremor-band power values, baseline asymmetry, and absolute changes for the baseline-defined more-affected limb. This table supports the primary descriptive IMU endpoint by providing participant-level values underlying the more-affected-limb analysis. Values are integrated 4–12 Hz acceleration power from wrist IMU acceleration, reported in (m/s^2^)^2^. For each participant, the table reports bilateral baseline values, identifies the baseline-defined more-affected and less-affected limbs, and provides the baseline asymmetry ratio. Condition values and absolute changes are reported for the baseline-defined more-affected limb; absolute change is condition value minus baseline value, with negative values indicating lower tremor-band power than baseline. Because the more-affected limb is defined from the same baseline recording used to calculate response, these values should be interpreted descriptively and with awareness of regression-to-the-mean risk; Table S2: Exploratory paired Wilcoxon signed-rank analyses of IMU outcomes. This table provides the complete exploratory statistical output referenced in the Results. Tests compare each intervention condition with baseline within metric and side using intervention-to-baseline ratios, with the baseline ratio defined as 1.0. Holm adjustment was applied across the three intervention comparisons within each metric-side stratum. Reported fields include sample size, median intervention-to-baseline ratio, median percent change, Wilcoxon signed-rank statistic, unadjusted *p*-value, and Holm-adjusted *p*-value. These analyses are secondary, exploratory, and hypothesis-generating because of the small sample size and fixed condition order, and they were not used to support confirmatory efficacy claims. W = Wilcoxon signed-rank statistic; Table S3: Participant flow and data availability. This table summarizes participant-level availability of the main laboratory and follow-up data streams used in the manuscript, including IMU recordings, digitized spiral data, HAB legibility, HAB time-to-complete, follow-up interview completion, and COPM availability. The final column documents reasons for missing, unavailable, or flagged data where applicable. P010 IMU data were unavailable because of an IMU file-generation error. P006 spiral data include a flagged left-hand LaceUp mean Δr value that matched the manually entered source record, but the original TRSPER export was not retrievable; that value is retained only as an unresolved source-data anomaly and is not used for interpretive conclusions.

## Abbreviations

The following abbreviations are used in this manuscript:

ADL	Activity of daily living
COPM	Canadian Occupational Performance Measure
DBS	Deep brain stimulation
ET	Essential tremor
HAB	Handwriting Assessment Battery for Adults
IMU	Inertial measurement unit
IRB	Institutional Review Board
LaceUp	Compression–weight sleeve evaluated in this pilot study
RMS	Root mean square
TAPS	Transcutaneous afferent patterned stimulation
TETRAS	The Essential Tremor Rating Assessment Scale
