# Supplementary material for: Feasibility Pilot of the LaceUp Compression–Weight Sleeve for Essential Tremor
Source: Bioengineering (Basel). 2026 Jul 8;13(7):785. doi: 10.3390/bioengineering13070785 (PMC13405488; doi:10.3390/bioengineering13070785)
Supplement: Supplementary file 1 [file bioengineering-13-00785-s001.zip › bioengineering-4385209-supplementary.pdf]

bioengineering

MDPI

Supplementary Materials

Table S1. Participant-level bilateral baseline 4–12 Hz tremor-band power values, baseline asymmetry, and absolute changes for the baseline-defined more-affected limb.

This table supports the primary descriptive IMU endpoint by providing participant-level values underlying the more-affected-limb analysis. Values are integrated 4–12 Hz acceleration power from wrist IMU acceleration, reported in $(m/s^2)^2$. For each participant, the table reports bilateral baseline values, identifies the baseline-defined more-affected and less-affected limbs, and provides the baseline asymmetry ratio. Condition values and absolute changes are reported for the baseline-defined more-affected limb; absolute change is condition value minus baseline value, with negative values indicating lower tremor-band power than baseline. Because the more-affected limb is defined from the same baseline recording used to calculate response, these values should be interpreted descriptively and with awareness of regression-to-the-mean risk.

Participant	More-affected limb	Baseline, more-affected limb	Less-affected limb	Baseline, less-affected limb	Baseline asymmetry ratio	Sleeve value	Sleeve absolute change	Wrist-weight value	Wrist-weight absolute change	LaceUp value	LaceUp absolute change
P001	L	4.918e-01	R	3.100e-02	15.87	9.440e-01	4.522e-01	1.03	5.353e-01	3.096e-01	-1.823e-01
P002	R	1.25	L	3.846e-01	3.24	1.20	-4.921e-02	7.359e-01	-5.120e-01	9.349e-01	-3.129e-01
P003	R	7.828e-01	L	2.417e-01	3.24	4.868e-01	-2.960e-01	3.471e-01	-4.357e-01	3.705e-01	-4.123e-01
P004	L	5.354e-01	R	3.397e-01	1.58	3.672e-01	-1.681e-01	4.231e-01	-1.123e-01	3.591e-01	-1.762e-01
P005	L	9.277e-01	R	8.267e-01	1.12	4.155e-01	-5.123e-01	6.178e-01	-3.099e-01	5.217e-01	-4.060e-01
P006	R	5.639e-01	L	4.121e-02	13.68	3.495e-01	-2.144e-01	4.212e-01	-1.427e-01	1.447e-01	-4.192e-01
P007	R	7.593e-01	L	3.830e-01	1.98	6.111e-01	-1.481e-01	3.351e-01	-4.242e-01	5.737e-01	-1.855e-01
P008	L	9.239e-02	R	6.022e-02	1.53	8.029e-02	-1.210e-02	8.614e-02	-6.243e-03	1.003e-01	7.889e-03
P009	L	1.078e-01	R	4.664e-02	2.31	2.088e-01	1.010e-01	1.146e-01	6.844e-03	1.972e-01	8.944e-02

Abbreviations: IMU, inertial measurement unit. Baseline asymmetry ratio = more-affected baseline value divided by less-affected baseline value.

Supplementary Table S2. Exploratory paired Wilcoxon signed-rank analyses of IMU outcomes.

This table provides the complete exploratory statistical output referenced in the Results. Tests compare each intervention condition with baseline within metric and side using intervention-to-baseline ratios, with the baseline ratio defined as 1.0. Holm adjustment was applied across the three intervention comparisons within each metric-side stratum. Reported fields include sample size, median intervention-to-baseline ratio, median percent change, Wilcoxon signed-rank statistic, unadjusted p-value, and Holm-adjusted p-value. These analyses are secondary, exploratory, and hypothesis-generating because of the small sample size and fixed condition order, and they were not used to support confirmatory efficacy claims. W = Wilcoxon signed-rank statistic.

Metric	Side	Comparison	n	Median ratio	Median %	W	p	Holm p
Tremor power (4–12 Hz)	L	Unweighted sleeve versus baseline	9	0.686	-31.4	21.0	0.9102	0.9102
Tremor power (4–12 Hz)	L	Wrist weights versus baseline	9	0.903	-9.7	13.0	0.3008	0.9023

Tremor power (4–12 Hz)	L	LaceUp versus baseline	9	0.629	-37.1	15.0	0.4258	0.9023
Tremor power (4–12 Hz)	R	Unweighted sleeve versus baseline	9	0.805	-19.5	19.0	0.7344	1.0000
Tremor power (4–12 Hz)	R	Wrist weights versus baseline	9	0.602	-39.8	11.0	0.2031	0.6094
Tremor power (4–12 Hz)	R	LaceUp versus baseline	9	0.756	-24.4	18.0	0.6523	1.0000
RMS jerk	L	Unweighted sleeve versus baseline	9	0.741	-25.9	13.0	0.3008	0.3008
RMS jerk	L	Wrist weights versus baseline	9	0.790	-21.0	5.0	0.0391	0.0781
RMS jerk	L	LaceUp versus baseline	9	0.708	-29.2	2.0	0.0117	0.0352
RMS jerk	R	Unweighted sleeve versus baseline	9	0.890	-11.0	14.0	0.3594	0.3594
RMS jerk	R	Wrist weights versus baseline	9	0.775	-22.5	4.0	0.0273	0.0820
RMS jerk	R	LaceUp versus baseline	9	0.893	-10.7	10.0	0.1641	0.3281

Abbreviations: IMU, inertial measurement unit; RMS, root mean square.

Table S3. Participant flow and data availability.

This table summarizes participant-level availability of the main laboratory and follow-up data streams used in the manuscript, including IMU recordings, digitized spiral data, HAB legibility, HAB time-to-complete, follow-up interview completion, and COPM availability. The final column documents reasons for missing, unavailable, or flagged data where applicable. P010 IMU data were unavailable because of an IMU file-generation error. P006 spiral data include a flagged left-hand LaceUp mean Δr value that matched the manually entered source record, but the original TRSPER export was not retrievable; that value is retained only as an unresolved source-data anomaly and is not used for interpretive conclusions.

**Participant**	**IMU**	**Spiral**	**HAB legibility**	**HAB time**	**Follow-up interview**	**COPM**	**Reason / verification notes**
P001	Avail.	Avail.	Avail.	NA	No	NA	HAB time-to-complete was not available; COPM was not being collected at this time of the study.
P002	Avail.	Avail.	Avail.	Avail.	Yes	NA	COPM was not being collected at this time of the study.
P003	Avail.	Avail.	Avail.	Avail.	Yes	NA	COPM was not being collected at this time of the study.
P004	Avail.	Avail.	Avail.	Avail.	No	NA	COPM was not being collected at this time of the study.
P005	Avail.	Avail.	Avail.	Avail.	No	NA	COPM was not being collected at this time of the study.
P006	Avail.	Flag	Avail.	Avail.	Yes	Yes	P006 left-hand LaceUp mean Δr value was checked against the manually entered source record and matched that record; original TRSPER export was not retrievable, so the value is retained only as an unresolved source-data anomaly and not interpreted.
P007	Avail.	Avail.	Avail.	Avail.	Yes	Yes	All listed laboratory and follow-up data available.
P008	Avail.	Avail.	NA	NA	No	NA	HAB legibility and time were not collected; COPM was not administered.

https://doi.org/10.3390/bioengineering13070785

P009	Avail.	Avail.	Avail.	Avail.	Yes	Yes	All listed laboratory and follow-up data available.
P010	Missing	Avail.	Avail.	Avail.	Yes	Yes	IMU data unavailable because of file-generation error

Abbreviations: COPM, Canadian Occupational Performance Measure; HAB, Handwriting Assessment Battery for Adults; IMU, inertial measurement unit.

Status shorthand: Avail. = available; NA = not administered/not applicable; Missing = unavailable data stream; Flag = verified source-data anomaly retained for transparency but not interpreted.
